# The Root Extract of *Peucedanum praeruptorum* Dunn Exerts Anticancer Effects in Human Non-Small-Cell Lung Cancer Cells with Different *EGFR* Mutation Statuses by Suppressing MET Activity

**DOI:** 10.3390/molecules27072360

**Published:** 2022-04-06

**Authors:** Hyun-Ji Park, Jae-Hoon Jeong, Shin-Hyung Park

**Affiliations:** Department of Pathology, College of Korean Medicine, Dong-Eui University, Busan 47227, Korea; 14554@deu.ac.kr (H.-J.P.); 15224@deu.ac.kr (J.-H.J.)

**Keywords:** *Peucedanum praeruptorum* Dunn, non-small-cell lung cancer, *EGFR* mutation, EGFR TKI resistance, apoptosis, MET

## Abstract

The aim of this study was to investigate the anticancer effects of the root extract of *Peucedanum praeruptorum* Dunn (EPP) in human non-small-cell lung cancer (NSCLC) cells and explore the mechanisms of action. We used four types of human lung cancer cell lines, including H1299 (*epidermal* *growth* *factor receptor* (*EGFR*) wild-type), PC9 (*EGFR* Glu746-Ala750 deletion mutation in exon 19; EGFR tyrosine kinase inhibitor (TKI)-sensitive), H1975 (*EGFR* L858R/T790M double-mutant; EGFR TKI-resistant), and PC9/ER (erlotinib-resistant) cells. EPP suppressed cell growth and the colony formation of NSCLC cells in a concentration-dependent manner. EPP stimulated chromatin condensation, increased the percentage of sub-G1 phase cells, and enhanced the proportion of annexin V-positive cells, demonstrating that EPP triggered apoptosis in NSCLC cells regardless of the *EGFR* mutation and EGFR TKI resistance status. The phosphorylation level of the signal transducer and activator of transcription 3 (STAT3) and AKT was decreased by EPP. The expression of STAT3 target genes was also downregulated by EPP. EPP reversed hepatocyte growth factor (HGF)-induced MET phosphorylation and gefitinib resistance. Taken together, our results demonstrate that EPP exerted anticancer effects not only in EGFR TKI-sensitive NSCLC cells, but also in EGFR TKI-resistant NSCLC cells, by suppressing MET activity.

## 1. Introduction

Lung cancer is the second most common cancer and the leading cause of cancer-related death worldwide [1]. It is estimated that 1,796,144 people died from lung cancer in 2020, which accounts for about 18% of all cancer-related deaths [1]. Although the prognosis of patients with lung cancer has consistently improved over the last decades, the 5-year survival rate of patients with lung cancer is still under 20% [1]. According to the statistics from USA, 57% of all lung cancer cases are diagnosed at advanced stages with metastatic disease, which contributes to the extremely poor 5-year survival rate (6%) in lung cancer patients with distant metastases [2]. Therefore, the development of novel strategies for early diagnosis and treatment is urgently needed.

*Epidermal growth factor receptor* (*EGFR*) mutations are identified in approximately 32% of non-small-cell lung cancer (NSCLC) patients. The prevalence of *EGFR* mutation is higher especially in Asians, females, and non-smokers [3]. The vast majority of *EGFR* mutations are either in-frame deletions in exon 19 (45%) or an L858R mutation in exon 20 (40%) [3]. These EGFR-activating mutations, also called EGFR-sensitizing mutations, make cancer cells become dependent upon the EGFR signaling pathway and stimulate cancer cell proliferation, angiogenesis, and the evasion of apoptosis by activating downstream effectors [4]. Advances in understanding the relevance of *EGFR* mutations in lung cancer progression have led to the development of EGFR-targeted therapies. Notably, EGFR tyrosine kinase inhibitors (TKIs), such as erlotinib or gefitinib, significantly improved the clinical outcomes of NSCLC patients with *EGFR* mutations [5,6,7,8]. However, a non-negligible percentage of cases showed a low response rate to EGFR TKIs due to the intratumoral *EGFR* heterogeneity in lung cancer [9,10]. Only cancer cells harboring *EGFR* mutations display responsiveness to EGFR TKIs, and the remaining non-mutated cancer cells that are insensitive to the treatment generate primary drug resistance [11,12]. In addition, acquired resistance eventually develops in patients who benefit from EGFR TKIs within one year of treatment. An *EGFR* T790M secondary mutation is the most common mechanism (60%) implicated in acquired resistance to EGFR TKIs [13]. Even though next-generation EGFR TKIs, such as osimertinib, have been developed to target the *EGFR* T790M mutation, additional resistance has been recognized [14]. The activation of accessory pathways, including MET, HER2, KRAS, PIK3CA, and BRAF, accounts for approximately 20% of the EGFR TKI resistance cases, suggesting that combining EGFR TKIs with other targeted agents can be a promising strategy to overcome TKI resistance [15]. However, so far, combination therapies have shown disappointing clinical outcomes and higher toxicity [15]. Therefore, new therapeutic drugs to conquer NSCLCs with intratumoral *EGFR* heterogeneity and overcome EGFR TKI resistance with minimum toxicity should be developed.

The root of *Peucedanum praeruptorum* Dunn (PP) has been traditionally used in eastern Asia for the treatment of coughs, asthma, and thick sputum [16]. According to traditional herbology, the PP root can reduce adverse Qi, resolve phlegm heat, and disperse wind heat [16]. Angular-type pyranocoumarins, including praeruptorin A, praeruptorin B, and praeruptorin E, have been recognized as the major active compounds in PP [17]. Previous studies revealed that the root extracts of PP reduced airway inflammation, which is related to its traditional use, treated cardiovascular diseases, and exerted antimicrobial effects [18,19,20,21]. More recently, Liang et al. reported that the methanolic extract of PP suppressed the proliferation of SGC7901 gastric cancer cells, which is the sole study demonstrating the anticancer activity of the crude PP extracts [22]. In the current study, we investigated whether the root extract of PP (EPP) exerted anticancer effects in human NSCLC cells with different *EGFR* mutation statuses and different sensitivities to EGFR TKIs and explored the molecular mechanisms.

## 2. Results

### 2.1. Suppression of Cell Growth by EPP in NSCLC Cells

**Figure 1:**
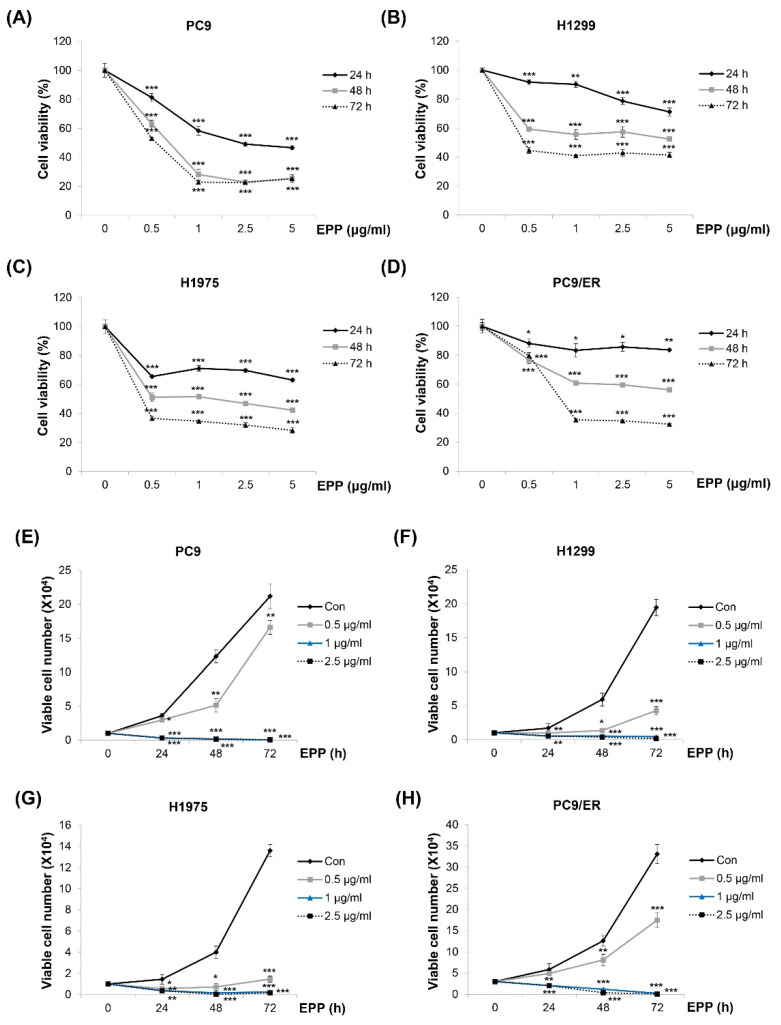
Effect of EPP on the growth of human NSCLC cell lines with different *EGFR* mutation statuses. (**A**–**D**) PC9 (*EGFR* Glu746-Ala750 deletion mutation in exon 19; EGFR TKI-sensitive, (**A**)), H1299 (*EGFR* wild-type, (**B**)), H1975 (*EGFR* L858R/T790M double-mutant; EGFR TKI-resistant, (**C**)), and PC9/ER (erlotinib-resistant, (**D**)) human NSCLC cells were incubated with EPP (0.5–5 μg/mL) for 72 h. Cell viability was evaluated using an MTT assay. (**E**–**H**) PC9 (**E**), H1299 (**F**), H1975 (**G**), and PC9/ER (H) cells were incubated with EPP (0.5–2.5 μg/mL) for different time durations (24–72 h). The number of cells was calculated using the trypan blue exclusion assay. Significance was determined using Student’s *t*-test (* *p* < 0.05, ** *p* < 0.01, *** *p* < 0.001 vs. untreated control). EPP, the root extract of *Peucedanum praeruptorum* Dunn; NSCLC, non-small-cell lung cancer; EGFR TKI, epidermal growth factor receptor tyrosine kinase inhibitor.

We investigated the effect of EPP on the cell growth of NSCLC cells with different *EGFR* mutation statuses. As displayed in Figure 1A–D, the viability of H1299 (*EGFR* wild-type), PC9 (*EGFR* Glu746-Ala750 deletion mutation in exon 19; EGFR TKI-sensitive), H1975 (*EGFR* L858R/T790M double-mutant; EGFR TKI-resistant), and PC9/ER (erlotinib-resistant) cells was decreased by EPP in a concentration- and time-dependent manner, which was measured by the MTT assay (Figure 1A–D). The results of the trypan blue exclusion assay also showed concentration and time-dependent reductions in cell proliferation following EPP treatment. Only 0.5 μg/mL of EPP was enough to exert anti-proliferative effects in NSCLC cells (Figure 1E–H). These results collectively demonstrate that EPP inhibited the cell growth of NSCLC cells regardless of the *EGFR* mutation status and the presence of EGFR TKI resistance.

### 2.2. Inhibition of Colony Formation by EPP in NSCLC Cells

**Figure 2:**
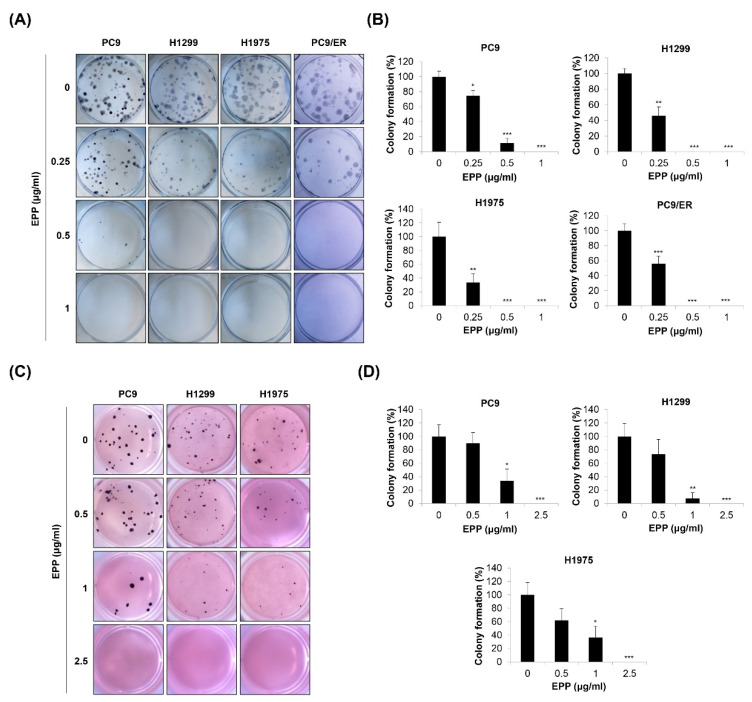
Effect of EPP on the colony formation of human NSCLC cell lines with different *EGFR* mutation statuses. H1299, PC9, H1975, and PC9/ER human NSCLC cells were seeded as a single-cell suspension for the anchorage-dependent colony formation assay (**A**,**B**) or the soft agar assay (**C**,**D**). The cells were incubated with different concentrations of EPP for 14 days. The colonies were photographed using a digital camera (**A**,**C**), and the number of colonies was counted (**B**,**D**). Significance was determined using Student’s *t*-test (* *p* < 0.05, ** *p* < 0.01, *** *p* < 0.001 vs. untreated control). EPP, the root extract of *Peucedanum praeruptorum* Dunn; NSCLC, non-small-cell lung cancer; EGFR, epidermal growth factor receptor.

Since cancer cells form colonies during tumorigenesis, we next investigated the effect of EPP on the colony formation of NSCLC cells with different *EGFR* mutation statuses. We found that the number of H1299, PC9, H1975, and PC9/ER cell colonies was gradually decreased in a concentration-dependent manner after 14 days of EPP treatment. As the duration of treatment was longer than that of the MTT assay or trypan blue exclusion assay, very low concentrations of EPP (0.25 μg/mL) significantly inhibited the colony formation of NSCLC cells (Figure 2A,B). To mimic the 3D tumorigenesis environment, we additionally performed a soft agar assay. Our results showed that EPP dose-dependently reduced the number of H1299, PC9, and H1975 cell colonies (Figure 2C,D). The colony formation tended to decrease following 0.5 μg/mL EPP treatment, even though this change was not significant due to the high standard deviation. EPP treatment at ≥ 1 μg/mL suppressed colony formation in the cell lines (Figure 2C,D). Taken together, our observations indicate that EPP diminished the colony-forming ability of NSCLC cells at very low concentrations regardless of the *EGFR* mutation status and the presence of EGFR TKI resistance. These results suggest that EPP might be applied at the early stage of cancer to suppress tumorigenesis.

### 2.3. Induction of Apoptosis by EPP in NSCLC Cells

**Figure 3:**
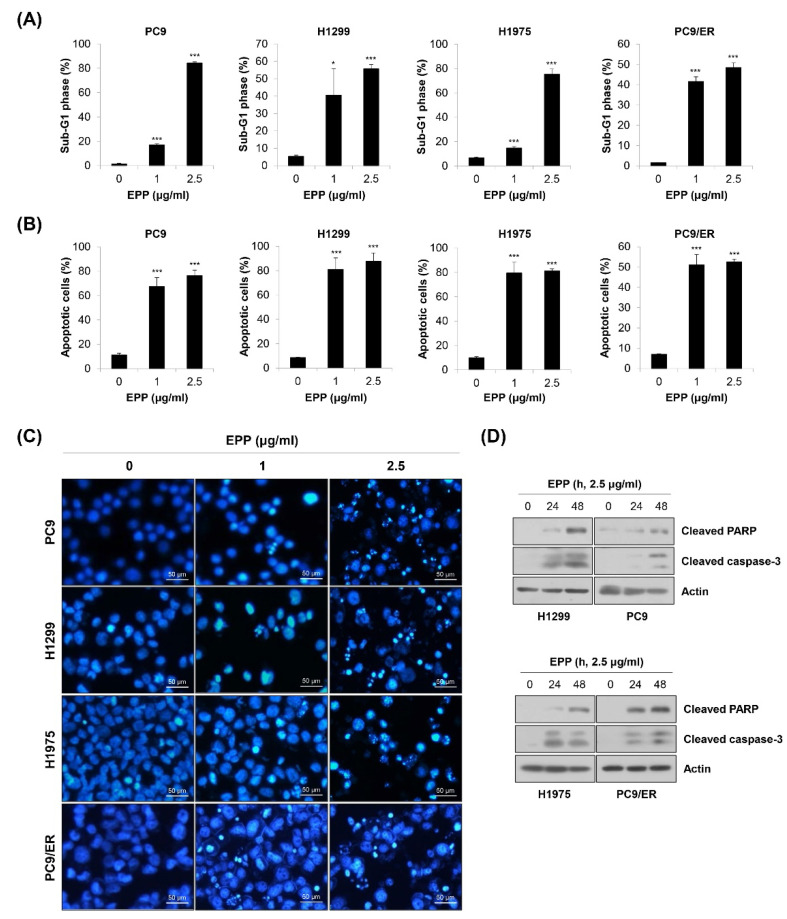
Induction of apoptosis by EPP in human NSCLC cell lines with different *EGFR* mutation statuses. (**A**–**C**) H1299, PC9, H1975, and PC9/ER human NSCLC cells were incubated with 1 μg/mL or 2.5 μg/mL EPP for 72 h. (**A**) The sub-G1 DNA content was measured via flow cytometry. (**B**) Following double-staining with annexin V and PI, the percentage of annexin V-positive cells, which indicated apoptotic cells, was evaluated via flow cytometry. Significance was determined using Student’s *t*-test (* *p* < 0.05, *** *p* < 0.001 vs. untreated control). (**C**) The DAPI staining assay was conducted to observe nuclear morphological changes (×200 magnification). The condensed and fragmented nuclei indicate apoptotic cells. Representative images of three independent experiments are shown. (**D**) Cells were incubated with 2.5 μg/mL EPP for different time durations (24 h or 48 h). The expression of cleaved caspase-3 and cleaved PAPR was assessed via Western blot analysis. Actin was used as the internal control. Representative images of duplicate experiments are shown. EPP, the root extract of *Peucedanum praeruptorum* Dunn; NSCLC, non-small-cell lung cancer; EGFR, epidermal growth factor receptor; PI, propidium iodide.

To investigate whether the above anti-proliferative and anti-colony formation effects of EPP were related to apoptosis induction, flow cytometry analysis was conducted. We observed that the percentage of cells with sub-G1 DNA content was dose-dependently increased in the four NSCLC cell lines (Figure 3A). We obtained similar results when apoptosis was detected using the annexin V-PI double-staining assay. The proportion of annexin V-positive cells was significantly elevated by 72 h of treatment with EPP (Figure 3B). We next performed the 4′,6-diamidino-2-phenylindole (DAPI) staining assay because nuclear morphological change is another marker of apoptosis. As displayed in Figure 3C, the number of cells with condensed and fragmented nuclei, a typical characteristic of apoptotic cells, was increased by EPP treatment in a concentration-dependent manner (Figure 3C). Consistently, the expressions of cleaved PARP and cleaved caspase-3, marker proteins of apoptosis, were upregulated following EPP treatment (Figure 3D). These results clearly indicate that EPP triggered apoptosis in NSCLC cells regardless of the *EGFR* mutation status and the presence of EGFR TKI resistance.

### 2.4. Inactivation of STAT3 and AKT by EPP in NSCLC Cells

**Figure 4:**
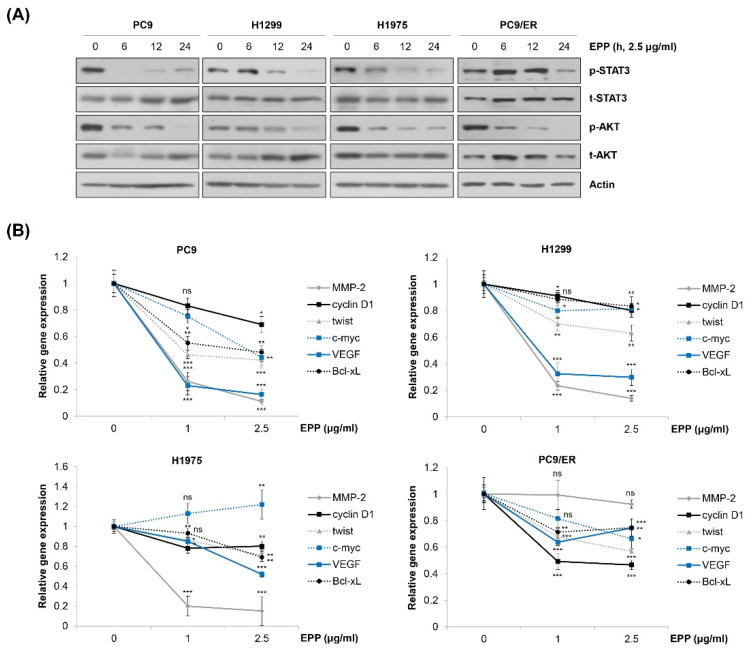
Inactivation of STAT3 and AKT by EPP in human NSCLC cell lines with different *EGFR* mutation statuses. (**A**) H1299, PC9, H1975, and PC9/ER human NSCLC cells were incubated with 2.5 μg/mL EPP for different time durations (6–24 h). Phosphorylated and total STAT3 and AKT proteins were detected via Western blot analysis. Actin was used as the internal control. Representative images of duplicate experiments are shown. (**B**) Cells were treated with 1 μg/mL or 2.5 μg/mL EPP for 48 h. The relative mRNA expression of STAT3 target genes was measured via real-time quantitative PCR. The data are expressed as the mean ± SD of triplicate experiments. Significance was determined using Student’s *t*-test (ns, not significant; * *p* < 0.05, ** *p* < 0.01, *** *p* < 0.001 vs. untreated control). EPP, the root extract of *Peucedanum praeruptorum* Dunn; NSCLC, non-small-cell lung cancer; EGFR, epidermal growth factor receptor; STAT3, signal transducer and activator of transcription 3.

We next explored the molecular mechanism by which EPP exerted anticancer activity in NSCLC cells. We observed that EPP induced apoptosis in both EGFR TKI-sensitive cells and EGFR TKI-resistant cells. Thus, we postulated several molecular candidates, including AKT and STAT3, which contribute not only to cancer cell proliferation but also to the emergence of EGFR TKI resistance [15,23,24,25,26]. Our results showed that both STAT3 and AKT were commonly dephosphorylated by EPP treatment in a time-dependent manner in the four NSCLC cell lines (Figure 4A). Consistently, the mRNA level of STAT3 target genes tended to decrease in EPP-treated cells, suggesting that EPP attenuated the transcriptional activity of STAT3 (Figure 4B). Changes in the expression of each target gene following EPP treatment varied depending upon the cell line, and several genes were not changed, while some were even slightly upregulated, by EPP treatment (Figure 4B). That may have been because the gene expressions were regulated not only by STAT3, but also by multiple transcription factors. Taken together, our results demonstrate that EPP suppressed the activity of both STAT3 and AKT in NSCLC cells regardless of the *EGFR* mutation status and the presence of EGFR TKI resistance.

### 2.5. Blockage of the MET Signaling Pathway by EPP in NSCLC Cells

**Figure 5:**
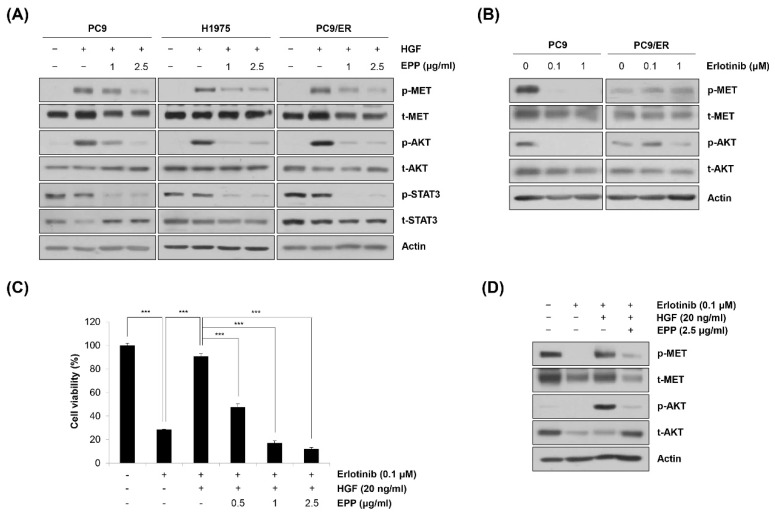
Blockage of MET by EPP in human NSCLC cell lines with different *EGFR* mutation statuses. (**A**) PC9, H1975, and PC9/ER human NSCLC cells were pretreated with 1 μg/mL or 2.5 μg/mL EPP for 24 h and stimulated with HGF (20 ng/mL) 30 min before harvest. Phosphorylated and total MET, AKT, and STAT3 proteins were detected via Western blot analysis. Representative images of duplicate experiments are shown. (**B**) PC9 and PC9/ER cells were treated with 0.1 μM or 1 μM erlotinib for 24 h. Phosphorylated and total MET and AKT proteins were detected via Western blot analysis. Representative images of duplicate experiments are shown. (**C**) PC9 cells were pretreated with EPP (0.5–2.5 μg/mL) for 2 h followed by co-treatment with erlotinib (0.1 μM) and HGF (20 ng/mL) for 72 h. Cell viability was measured using the MTT assay. The data are expressed as the mean ± SD of triplicate experiments. Significance was determined using Student’s *t*-test (*** *p* < 0.001 vs. respective control). (**D**) PC9 cells were co-treated with EPP (2.5 μg/mL) and erlotinib (0.1 μM) for 24 h and stimulated with HGF (20 ng/mL) 30 min before harvest. Phosphorylated and total MET and AKT proteins were detected using Western blot analysis. Representative images of duplicate experiments are shown. EPP, the root extract of *Peucedanum praeruptorum* Dunn; NSCLC, non-small-cell lung cancer; EGFR, epidermal growth factor receptor; HGF, hepatocyte growth factor; STAT3, signal transducer and activator of transcription 3.

We next investigated the phosphorylation level of MET, a probable upstream kinase of STAT3 and AKT [27]. As shown in Figure 5A, hepatocyte growth factor (HGF) stimulated the phosphorylation of MET, which was prevented by pretreatment with EPP. The phosphorylation level of AKT also showed the same pattern as that of MET, indicating that MET was involved in regulating AKT activity (Figure 5A). However, HGF did not phosphorylate STAT3, suggesting that STAT3 activity might be regulated by a MET-independent mechanism (Figure 5A). In addition, HGF-induced phosphorylation of MET was not inhibited by EPP in *EGFR* wild-type H1299 cells, indicating that the upstream target of EPP that regulates the activity of STAT3 and AKT might be different according to the cell type (Supplementary Materials Figure S1). As *MET* amplification and protein hyperactivation are important resistance mechanisms of EGFR-targeted therapies, we hypothesized that EPP could be applied to overcome EGFR TKI resistance [15,28]. Notably, we found that neither MET nor AKT was dephosphorylated following erlotinib treatment in PC9/ER cells, while their activity was totally suppressed by erlotinib in PC9 cells (Figure 5B). These results suggest that MET activation might be implicated in erlotinib resistance in PC9/ER cells. To determine whether MET activity was important in generating EGFR TKI resistance and whether EPP could suppress erlotinib resistance, we conducted an MTT assay. As shown in Figure 5C, HGF treatment caused erlotinib resistance in PC9 cells, which was reversed by combined treatment with erlotinib and EPP (Figure 5C). The activation of the MET/AKT pathway was involved in HGF-induced erlotinib resistance, and EPP significantly suppressed the phosphorylation of MET and AKT in PC9 cells (Figure 5D). Taken together, these results demonstrate that EPP exerted anticancer effects not only in EGFR TKI-sensitive cells but also in EGFR TKI-resistant cells by inhibiting the MET signaling pathway.

### 2.6. Identification of Specific Compounds Contributing to the Anticancer Effect of EPP

High-performance liquid chromatography–mass spectrometry (HPLC-MS) analysis was conducted to identify praeruptorin A (PA), a coumarin generally found in the roots of PP, in EPP [29]. The total chromatograms of PA and EPP were acquired at 330 nm via UV detection. We found that the peak of PA was detected at a retention time of 25.95 min (Supplementary Figure S2 and Table 1). The chromatogram of EPP also included a peak at a retention time of 25.94 min. Moreover, the molecular weight of the EPP peak detected via MS analysis was the same as that of PA at *m*/*z* 404.1 [M + H]^+^, indicating that PA was contained in EPP (Supplementary Figure S2 and Table 1). As PA can be used for the standardization of the PP root, our results demonstrate that a standardized PP specimen was used in this study.

**Figure 6:**
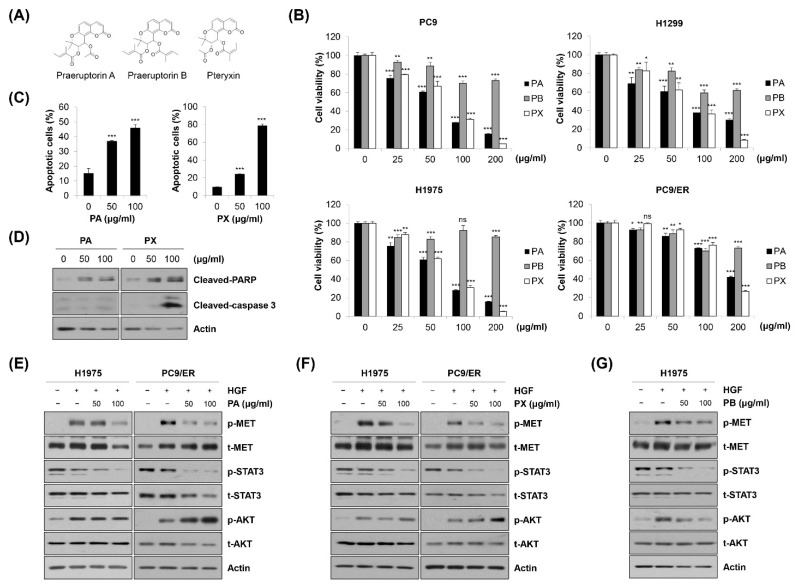
Identification of specific compounds contributing to the anticancer effect of EPP. (**A**) The chemical structures of praeruptorin A (PA), praeruptorin B (PB), and pteryxin (PX) are shown. (**B**) PC9, H1299, H1975, and PC9/ER human NSCLC cells were treated with PA, PB, and PX for 72 h. Cell viability was evaluated using the MTT assay. (**C**,**D**) H1975 cells were treated with either PA or PX for 72 h. (**C**) The percentage of annexin V-positive cells was evaluated via flow cytometry. Significance was determined using Student’s *t*-test (ns, not significant; * *p* < 0.05, ** *p* < 0.01, *** *p* < 0.001 vs. untreated control). (**D**) The expression of cleaved caspase-3 and cleaved PAPR was assessed via Western blot analysis. (**E**,**F**) H1975 and PC9 cells were pretreated with either PA (**E**) or PX (**F**) for 24 h and stimulated with HGF (20 ng/mL) 30 min before harvest. (**G**) H1975 cells were pretreated with PB for 24 h and stimulated with HGF (20 ng/mL) 30 min before harvest. (**E**–**G**) Phosphorylated and total MET, AKT, and STAT3 proteins were detected using Western blot analysis. Actin was used as the internal control. Representative images of duplicate experiments are shown. NSCLC, non-small-cell lung cancer; HGF, hepatocyte growth factor; STAT3, signal transducer and activator of transcription 3.

We further investigated which constituents in EPP contributed to the anticancer activity of EPP. PA, praeruptorin B (PB), and pteryxin (PX), coumarins contained in the PP root, were treated in NSCLC cells. The chemical structures are shown in Figure 6A. As shown in Figure 6B, the cell viability was significantly decreased by PA or PX treatment in a concentration-dependent manner, while the growth-inhibitory effect of PB in these cell lines was relatively marginal. Both PA and PX increased the proportion of annexin V-positive cells and upregulated the cleavage of PARP in H1975 cells, suggesting that PA and PX induced apoptosis (Figure 6C,D). However, the expression of cleaved caspase-3 was increased only by PX, but not by PA, indicating that the mechanism by which PA and PX induced apoptosis might be different (Figure 6D). Next, we conducted Western blot analysis to evaluate the effects of PA and PX on the activity of the MET signaling pathway. We found that both PA and PX suppressed the HGF-induced phosphorylation of MET in H1975 and PC9/ER cells (Figure 6E,F). Even though STAT3 was not activated by HGF, it was clearly dephosphorylated by PA or PX treatment, which was consistent with the results in Figure 5A (Figure 6E,F). However, neither PA nor PX inhibited the HGF-stimulated phosphorylation of AKT, suggesting that other constituents in EPP would suppress AKT activity (Figure 6E,F). Interestingly, AKT was dephosphorylated by PB, which was paralleled by a slight decrease in phospho-MET (Figure 6G). Taken together, our observations suggest that multiple constituents in EPP, including PA, PB, and PX, collaborate with each other to contribute to the anticancer activity of EPP in NSCLC cells.

## 3. Discussion

In this study, we investigated the anticancer mechanism of EPP in NSCLC cells. We found that EPP suppressed cell proliferation and colony formation and induced apoptosis in NSCLC cells regardless of the *EGFR* mutation status. As the genetic heterogeneity of lung tumors is responsible for the resistance or sensitivity to therapeutic drugs, EPP, which exerts general anticancer effects in cells with different *EGFR* mutations, could be beneficial for managing NSCLC [9,10]. In addition, not only EGFR TKI-sensitive cells, but also EGFR TKI-resistant cells with an *EGFR* T790M secondary mutation or MET activation showed high sensitivity to EPP treatment, with an IC50 of less than 1 μg/mL. We found that EPP suppressed the activity of STAT3 and AKT, pivotal regulators of cancer cell survival and growth, suggesting that these molecules could mediate the anticancer effects of EPP [25,26].

We postulated that MET was a target of EPP because of the following reasons. First, MET is a well-known tyrosine kinase receptor that activates both STAT3 and AKT [27]. As EPP treatment significantly dephosphorylated STAT3 and AKT in NSCLC cells, we searched for probable upstream kinases that regulated the activity of STAT3 and AKT. Second, MET stimulates cancer cell proliferation, invasion, and angiogenesis [30]. Many studies have demonstrated that the aberrant activation of the MET pathway was closely related to the poor prognosis of cancer patients, making MET an attractive target for cancer therapy [31]. Third, MET is a critical mediator of EGFR TKI resistance. Although the *EGFR* T790M secondary mutation is recognized as the most common mechanism, *MET* amplification is another critical factor that accounts for 5–22% of the resistance cases in NSCLC patients treated with first-generation EGFR TKIs [15,28]. As EPP exerted anticancer activity in EGFR TKI-resistant cells, we hypothesized that the molecules implicated in EGFR TKI resistance could be regulated by EPP. In addition, MET phosphorylation, which was totally decreased by erlotinib in naïve PC9 cells, was not altered by erlotinib in PC9/ER cells, suggesting that the MET signaling pathway could be involved in erlotinib resistance in PC9/ER cells. Our results clearly showed that the HGF-induced activation of MET was significantly decreased by EPP treatment. Interestingly, the phosphorylation level of AKT showed the same pattern as that of MET, indicating that AKT activity was regulated by MET. However, STAT3 was not activated by HGF, implying that another upstream kinase, such as interleukin (IL)-6 receptor/Janus kinases (JAKs) or Src, could mediate STAT3 activation [25]. We also found that combined EPP and erlotinib treatment prevented HGF-mediated erlotinib resistance in PC9 cells, which demonstrated that EPP could overcome EGFR TKI resistance by blocking MET/AKT activity. Previous studies reported that MET amplification-induced PI3K/AKT activation contributed to the acquired resistance to first-generation EGFR TKIs, suggesting that the MET/AKT pathway was involved in erlotinib resistance [30]. Consistently, the inhibition of AKT significantly diminished HGF-mediated erlotinib resistance in NSCLC cells, which supports our results [32].

To the best of our knowledge, this was the first study demonstrating the anticancer effects of the crude extract of PP in NSCLC cells. The reasons why investigating the effects of the crude extract of PP is important are as follows. First, PP has been used in traditional Oriental medicine (TOM) as a whole extract, not as a purified single compound. Second, as the crude extract contains a variety of constituents, its activity can derive from the complex interaction between the compounds. Even if a certain compound exerts anticancer activity, the other constituents can counteract it. Likewise, even if a certain constituent shows only slight anticancer activity, the effect can be reinforced by a synergistic effect with other components. Several constituents of PP, including PA and PB, have been recognized to inhibit cancer cell proliferation and invasion [33,34]. So far, there has been no study reporting the anticancer activity of PX. In addition, no constituent of PP has been reported to regulate MET activity. Our results showed that both PA and PX, but not PB, significantly attenuated cell proliferation and induced apoptosis in NSCLC cells by deactivating MET and STAT3. However, neither PA nor PX inhibited AKT activation. We found that PB significantly suppressed the phosphorylation of AKT, which has been also demonstrated by a previous study [34]. Thus, our results collectively suggest that multiple constituents in EPP would collaborate with each other to contribute to the anticancer activity of EPP in NSCLC cells.

Taken together, our results demonstrate that EPP exerted anticancer activity in NSCLC cells regardless of the *EGFR* mutation status and the presence of EGFR TKI resistance. As EPP suppressed the STAT3 and MET/AKT pathways, which stimulate tumor growth and confer EGFR TKI resistance, EPP could be applied not only to EGFR TKI-naïve or -sensitive NSCLC, but also to EGFR TKI-resistant NSCLC. More studies are warranted to validate the anticancer effects of EPP and determine the specific compound(s) mediating its activity.

## 4. Materials and Methods

### 4.1. Preparation of EPP

The root of PP originating from Guizhou (China) on April 23, 2021, was purchased from Bonchomaru (Seoul, Korea). The authentication of the sample was conducted by Deokin Pharmaceutical Co., Ltd. (Seoul, Korea). The voucher sample (#21152-01) was deposited in the herbarium of the Pathology Laboratory at the College of Korean Medicine, Dong-eui University, Busan, Korea. The root of PP (50 g) was extracted with 800 mL of 80% ethyl alcohol in a shaking incubator at 40 °C for 48 h. Once the first extract was collected, 300 mL of 80% ethyl alcohol was added again to the PP root and incubated for another 24 h in a second extraction. The mixture of the first and second extracts then underwent a concentration step using a vacuum rotary evaporator under reduced pressure. After the concentrated extract was lyophilized for 3 days, 12.53 g of EPP powder was obtained (yield = 25.06%). The EPP powder was then reconstituted in dimethyl sulfoxide (DMSO; Amresco, Solon, OH, USA) at 200 mg/mL, and was diluted to the working concentration prior to immediate use.

### 4.2. HPLC-MS Analysis

HPLC analysis was conducted using a Dionex UltiMate 3000 UHPLC system (Thermo Fisher Scientific, San Jose, CA, USA) and Thermo Chromeleon 7 software (Thermo Fisher Scientific, San Jose, CA, USA). Praeruptorin A (ChemFaces, Wuhan, China) and EPP powder were dissolved in 50% methanol at final concentrations of 20 mg/mL and 50 μg/mL, respectively. Separation was performed on a YMC Triart C18 column (150 × 2.0 mm, 3 μm) using distilled water (DW) and acetonitrile as solvent A and solvent B, respectively. The mobile conditions were as follows: 80% solvent A and 20% solvent B initially, 10% solvent A and 90% solvent B for 30 min, 10% solvent A and 90% solvent B for 5 min, 80% solvent A and 20% solvent B for 0.5 min, and 80% solvent A and 20% solvent B for 9.5 min as the final conditions. The flow rate was 0.2 mL/min, and the column temperature was 25 °C. The detection wavelength was 330 nm. MS analysis was performed using a Compact mass spectrometer LC-MS system (Advion, Ithaca, NY, USA). The mass spectra were recorded over *m*/*z* 100–1200 in the positive electrospray ionization (ESI) mode.

### 4.3. Cell Culture

PC9, H1299, and H1975 human NSCLC cell lines were provided by Professor Ho-Young Lee (Seoul National University, Seoul, Korea). The cells were cultured in RPMI-1640 medium (WelGENE, Daegu, Korea) supplemented with 10% fetal bovine serum (FBS; WelGENE) and 1% antibiotics (WelGENE) and maintained at 37 °C under 5% CO_2_ conditions. The erlotinib-resistant PC9 (PC9/ER) cell line was established as described in our previous study [35]. PC9/ER cells were cultured in RPMI-1640 medium containing 25 μM erlotinib, and the other culture conditions were the same as those mentioned above.

### 4.4. MTT Assay

Cells were seeded at a density of 3 × 10^3^ cells/well in 96-well plates. After overnight stabilization, the cells were treated with either EPP (0.5–5 μg/mL) or its constituents, including PA, PB (ChemFaces), and PX (ChemFaces), for 72 h. Subsequently, MTT (3-(4,5-dimethylthiazol-2-yl)-2,5-diphenyltetrazolium bromide; Duchefa, Haarlem, The Netherlands) solution (4 mg/mL) was added to the culture medium at a final concentration of 0.4 mg/mL and incubated for an additional 2 h. The culture medium was then carefully discarded and the insoluble MTT formazan was solubilized by adding 100 μL of DMSO. Cell viability was evaluated by measuring the absorbance of each well at 540 nm using a microplate reader (SpectraMax M3; Molecular Devices, San Jose, CA, USA).

### 4.5. Trypan Blue Exclusion Assay

Cells were plated at a density of 2 × 10^4^ cells/well in 12-well plates. After overnight stabilization, the cells were challenged with different concentrations of EPP (0.5–2.5 μg/mL). The cells were collected at 24 h, 48 h, and 72 h post-treatment with EPP, and suspended in phosphate-buffered saline (PBS; Donginbio, Seoul, Korea). The cell suspension was then mixed with the same volume of 0.4% trypan blue solution (WelGENE). Blue-colored cells were considered to be dead cells, and the number of colorless live cells was counted using a hemocytometer.

### 4.6. Colony Formation Assay

For the anchorage-dependent colony formation assay, cells were plated as a single-cell suspension at a density of 2 × 10^2^ cells/well in 12-well plates. After overnight stabilization, the cells were challenged with different concentrations of EPP (0.25–1 μg/mL) and were incubated for 14 days until the colonies were fully grown. The culture medium containing EPP was replaced every three days. The colonies were fixed with methanol for 5 min, stained with hematoxylin (Sigma-Aldrich, St. Louis, MO, USA) for 30 min, and washed several times with distilled water (DW). All procedures were conducted at room temperature. The anchorage-independent colony-forming ability of cancer cells was evaluated using the soft agar assay. First, 1% bottom agar, made by diluting 4% SeaPlaque agarose (Lonza, Rockland, ME, USA) with warm culture media, was overlaid on 24-well plates and solidified at room temperature for 1 h. Then, 0.4% top agar containing 1 × 10^3^ cells was added onto the bottom agar. After the top agar was completely solidified at room temperature, 0.5 mL of warm culture media containing different concentrations of EPP (0.5–2.5 μg/mL) was added onto the top agar. At 14 days post-treatment, the colonies were stained by adding MTT solution to the culture medium at 0.5 mg/mL and incubated at 37 °C for 1.5 h. The culture medium was then replaced with PBS, and images of the colonies were taken with a digital camera (Canon, Tokyo, Japan).

### 4.7. Flow Cytometry

Cells were plated at a density of 1 × 10^5^ cells/well in 6-well plates. After overnight stabilization, the cells were treated with 1 μg/mL or 2.5 μg/mL EPP for 72 h. For cell cycle analysis, the cells were fixed with 80% ethyl alcohol at 4 °C for 1 h and stained for 30 min with 50 µg/mL propidium iodide (PI) solution (Sigma-Aldrich) supplemented with 30 µg/mL RNase A (Sigma-Aldrich). The cells were then washed and resuspended in PBS. The cell cycle distribution was analyzed by flow cytometry (FACSCaliber, Becton Dickinson and Company, San Jose, CA, USA). The percentage of cells with sub-G1 DNA content, considered to be an apoptotic population, was measured using CellQuest software. The apoptotic cells were also detected using the annexin V-PI double-staining assay. Cells treated with EPP (1 μg/mL or 2.5 μg/mL) for 72 h were stained with the Annexin V-FITC Apoptosis Detection Kit I (BD Biosciences Pharmingen, San Diego, CA, USA) according to the manufacturer’s instructions. The stained cells were analyzed via flow cytometry (FACSCaliber, Becton Dickinson and Company), and the percentage of the annexin V-positive cells was measured using CellQuest software.

### 4.8. DAPI Staining

Cells were plated at a density of 1 × 10^5^ cells/well in 6-well plates. After overnight stabilization, the cells were treated with 1 μg/mL or 2.5 μg/mL EPP for 72 h. The cells were then fixed with 3.7% paraformaldehyde (Sigma-Aldrich) for 30 min at 4 °C and resuspended in 200 μL of PBS. Then, the cells were attached to slide glasses using a Cytospin (Shandon Inc., Pittsburgh, PA, USA). For nuclear staining, the cells were stained for 10 min in the dark with 4,6-diamidino-2-phenylindole-dihydrochloride (DAPI) solution at a final concentration of 2.5 μg/mL. After being washed twice with PBS, the cells were mounted with mounting medium (Biomeda, Foster City, CA, USA), and the nuclei were observed under a fluorescence microscope (Carl Zeiss AG, Oberkochen, Germany) at ×200 magnification.

### 4.9. Western Blot Analysis

Cells were lysed with cold RIPA buffer (Thermo Fisher Scientific) with an added protease inhibitor cocktail (Thermo Fisher Scientific) and phosphatase inhibitors (1mM Na_3_VO_4_ and 100 mM NaF). The protein concentration of each sample was measured using the bicinchoninic acid (BCA) protein assay kit (Pierce Biotechnology, Rockford, IL, USA). Protein was loaded (20 µg per lane) and separated by 8–12% sodium dodecyl sulfate (SDS)–polyacrylamide gel electrophoresis (PAGE). The proteins were then transferred onto polyvinylidene fluoride (PVDF) membranes (Millipore, Bedford, MA, USA). The membrane was blocked with 3% bovine serum albumin (BSA, GenDEPOT, Barker, TX, USA) for 30 min and was subjected to immunoblotting using the indicated primary antibodies at 1:1000 dilutions at 4 °C overnight. Following several washing steps with Tris-buffered saline (TBS) supplemented with 0.1% Tween-20 (TBST), the membrane was probed with the secondary antibody (1:10,000 dilution) for 1 h at room temperature. Specific signals were detected using the D-Plus ECL Femto System (Donginbio, Seoul, Korea). The intensity of each blot was quantified using ImageJ software (version 1.52a; National Institutes of Health, Bethesda, MD, USA). All primary antibodies except for the actin antibody (Santa Cruz Biotechnology, Santa Cruz, CA, USA) were purchased from Cell Signaling Technology (Beverly, MA, USA), and the corresponding anti-mouse and anti-rabbit secondary antibodies were purchased from Bethyl Laboratories (Montgomery, TX, USA) and Enzo Life Sciences (Farmingdale, NY, USA), respectively.

### 4.10. Reverse Transcription–Quantitative Polymerase Chain Reaction (RT-qPCR)

Total RNA was extracted using TRIzol reagent (Invitrogen; Thermo Fisher Scientific) and was quantified using a microplate reader (SpectraMax M3; Molecular Devices). Total RNA (1 μg) was used to synthesize first-strand cDNA using the PrimeScript RT reagent kit (Takara, Shiga, Japan). The cDNA templates were diluted 50-fold in nuclease-free water and subjected to quantitative real-time PCR analysis performed on a CFX Connect Real-Time PCR Detection System (Bio-Rad Laboratories, Hercules, CA, USA) using SYBR green (Enzynomics, Daejeon, Korea). The primer sequence and annealing temperature for each gene are shown in Table 2.

### 4.11. Statistical Analysis

Each result is expressed as the mean ± standard deviation (SD) of three independent experiments. The statistical analysis was performed using a paired Student’s *t*-test. Differences at *p* < 0.05 were considered to be statistically significant.

## Figures and Tables

**Table 1:** The HPLC-MS data of PA and EPP peak.

Name	RT ^1^	MS ^2^ [M + H]^+^ (*m*/*z*)
PA ^3^	25.95	404.0
EPP ^4^ peak	25.94	404.1

^1^ RT: retention time, ^2^ MS: mass spectrometry, ^3^ PA: praeruptorin A, ^4^ EPP: the root extract of *Peucedanum praeruptorum* Dunn.

**Table 2:** Primer sequence and annealing temperature for each gene.

Name	Primer Sequence (5′→3′)	AT ^1^ (°C)
MMP-2	Forward: CGC ATC TGG GGC TTT AAA CAT Reverse: CCA TTA GCG CCT CCA TCG TA	60
Cyclin D1	Forward: CCT GTC CTA CTA CCG CCT CA Reverse: TCC TCC TCT TCC TCC TCC TC	55
Twist	Forward: TGT CCG CGT CCC ACT AGC Reverse: TGT CCA TTT TCT CCT TCT CTG GA	55
c-myc	Forward: CTT CTC TCC GTC CTC GGA TTC T Reverse: GAA GGT GAT CCA GAC TCT GAC CTT	55
VEGF	Forward: AGG AGG AGG GCA GAA TCA TCA Reverse: CTC GAT TGG ATG GCA GTA GCT	55
Bcl-xL	Forward: GTT CCC TTT CCT TCC ATC C Reverse: TAG CCA GTC CAG AGG TGA G	55
Actin	Forward: ACT ACC TCA TGA AGA TC Reverse: GAT CCA CAT CTG CTG GAA	55

^1^ AT: annealing temperature.

## Data Availability

The data presented in this study are available on request from the corresponding author.

## Supplementary Materials

The following supporting information can be downloaded at: https://www.mdpi.com/article/10.3390/molecules27072360/s1, Figure S1: Effect of EPP on the activity of MET signaling pathway in EGFR wild-type H1299 cells. Figure S2: Identification of praeruptorin A from EPP by HPLC-MS analysis.

## Sample Availability

Samples of the root extract of *Peucedanum praeruptorum* Dunn (EPP) are available from the authors.
