# Supplementary material for: The Root Extract of Peucedanum praeruptorum Dunn Exerts Anticancer Effects in Human Non-Small-Cell Lung Cancer Cells with Different EGFR Mutation Statuses by Suppressing MET Activity
Source: Molecules. 2022 Apr 6;27(7):2360. doi: 10.3390/molecules27072360 (PMC9000538; doi:10.3390/molecules27072360)
Supplement: Supplementary file 1 [file molecules-27-02360-s001.zip › molecules-1603743-supplementary.pdf]

Supplementary Materials

# The Root Extract of *Peucedanum praeruptorum* Dunn Exerts Anticancer Effects in Human Non-Small-Cell Lung Cancer Cells with Different *EGFR* Mutation Statuses by Suppressing MET Activity

**Hyun-Ji Park †, Jae-Hoon Jeong and Shin-Hyung Park †,***

Department of Pathology, College of Korean Medicine, Dong-Eui University, Busan 47227, Korea; 14554@deu.ac.kr (H.-J.P.); 15224@deu.ac.kr (J.-H.J.)

* Correspondence: omdpark@deu.ac.kr; Tel.: +82-51-890-3332

† These authors contributed equally to this work.

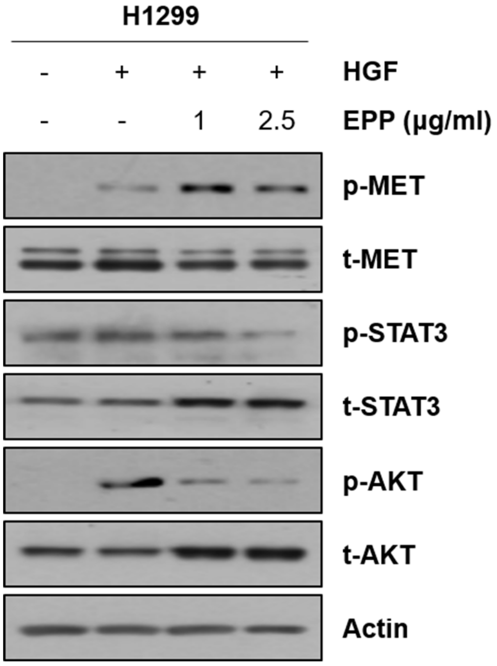


**Supplementary Figure S1. Effect of EPP on the activity of MET signaling pathway in *EGFR* wild-type H1299 cells.** H1299 human NSCLC cells were pretreated with 50 μg/mL or 100 μg/mL EPP for 24 h and stimulated with HGF (20 ng/mL) 30 min before harvest. Phosphorylated and total MET, AKT, and STAT3 proteins were detected by Western blot analysis. Actin was used as the internal control. Representative images of duplicate experiments are shown. EPP, the root extract of *Peucedanum praeruptorum* Dunn; NSCLC,

non-small-cell lung cancer; EGFR, epidermal growth factor receptor; HGF, hepatocyte growth factor; STAT3, signal transducer and activator of transcription 3.

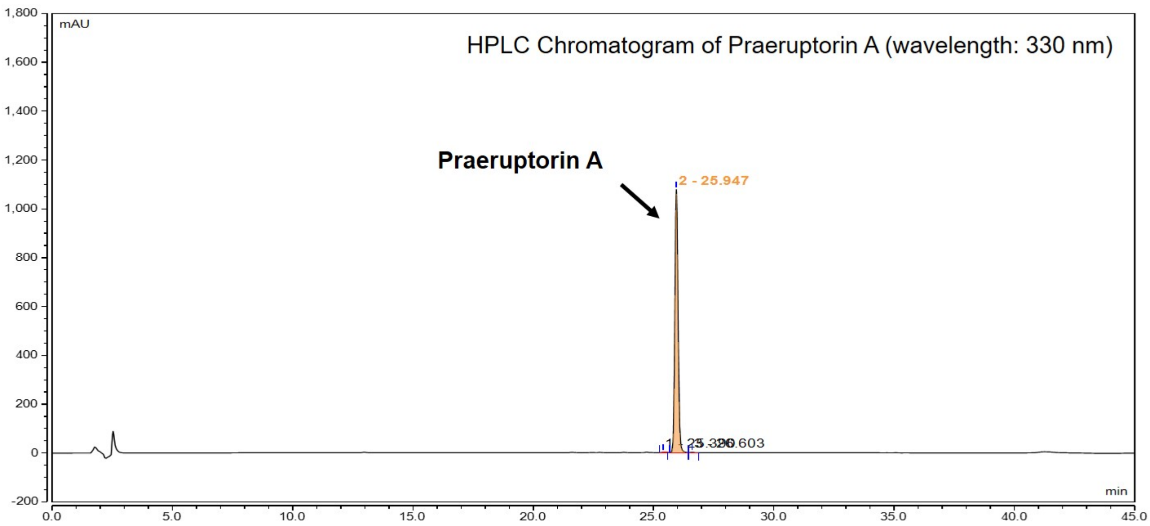


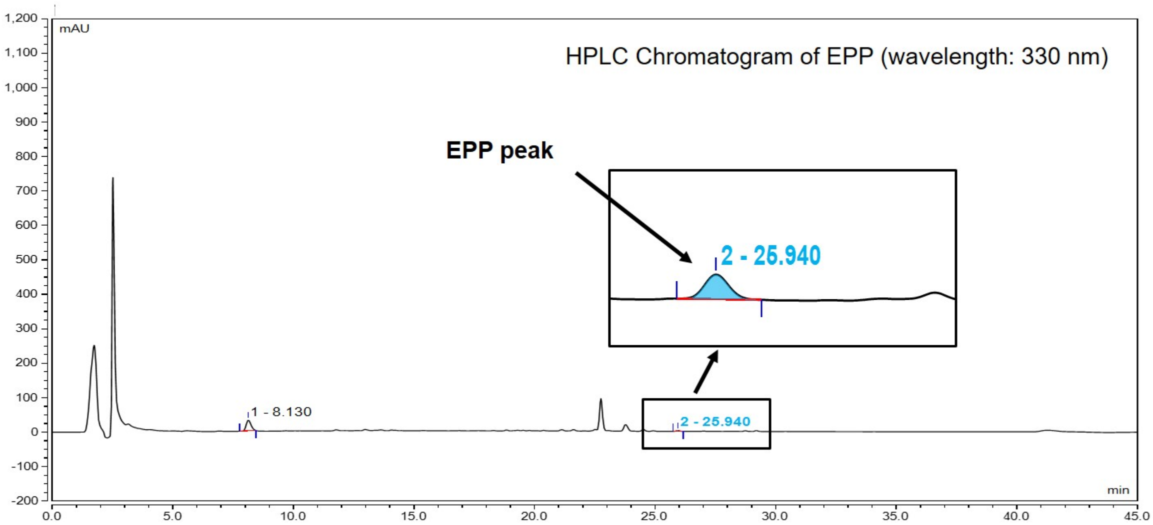

**Supplementary Figure S2. Identification of praeruptorin A from EPP by HPLC-MS analysis.** Total HPLC chromatograms of praeruptorin A (A) and EPP (B) were obtained at 330 nm. EPP, the root extract of *Peucedanum praeruptorum* Dunn; HPLC-MS, high-performance liquid chromatography-mass spectrometry.
